# Revisiting the Surgical Approaches to Decompression in Quadrilateral Space Syndrome: A Cadaveric Study

**DOI:** 10.7759/cureus.22619

**Published:** 2022-02-26

**Authors:** Sundip Charmode, Simmi Mehra, Sudhir Kushwaha

**Affiliations:** 1 Anatomy, All India Institute of Medical Sciences, Rajkot, Rajkot, IND; 2 Orthopaedics, All India Institute of Medical Sciences, Gorakhpur, Gorakhpur, IND

**Keywords:** posterior circumflex humeral artery, quadrangular space, axillary nerve, decompression, surgical approach

## Abstract

Background

Quadrangular space syndrome involves compression of the axillary nerve and posterior circumflex humeral artery. In a few cases, its management requires surgical decompression. The current study reviews the surgical approaches used in the decompression of neurovascular structures and presents our reflections and recommendations.

Methodology

In this study, four human cadavers were used for dissection of the axillae and the scapular region by the senior residents of the Department of Anatomy and Department of Orthopedics. The residents dissected the quadrangular space in the eight upper limbs using anterior and posterior surgical approaches.

Results

To identify the quadrangular space and secure its contents, the posterior approach was recognized as the easier and quicker method by both Anatomy and Orthopedic residents; however, it may result in increased postoperative morbidity. Although the anterior (deltopectoral) approach involves more skill, it reduces postoperative morbidity.

Conclusions

The anterior (deltopectoral) approach with suggested modifications can be an effective method in the surgical decompression of quadrangular space syndrome. The authors suggest more cadaveric studies to provide anatomists and surgeons with the opportunity to practice and evaluate older and newer surgical approaches.

## Introduction

Quadrilateral space syndrome (QSS) is an uncommon neurovascular entrapment condition involving the axillary nerve (AXN) and/or posterior humeral circumflex course (PHCA) in the quadrilateral space due to injury, fibrous bands, or hypertrophy of the muscular border [1]. The management of QSS involves a decompression procedure with various approaches, which remain unevaluated, especially in the Indian population.

The syndrome usually affects the dominant arm of young 20-35-year-old adults, particularly athletes involved in overhead sports, such as volleyball [2], baseball [3], swimming [4], and other activities with frequent abduction and external rotation, such as yoga [5] or window cleaning [6]. Neurogenic QSS presents with paraesthesia, fasciculations, weakness, or neurogenic pain in a non-specific manner. Indications for intense ischemia reminiscent of vascular QSS include pain, pallor, absent pulses, thrombosis, or embolism (coolness or cyanosis of the hands or digits). Notwithstanding vascular and neurogenic indications, patients with QSS suffer from muscular atrophy and associated weakness due to denervation [7-9].

The intermuscular space is bordered superiorly by subscapularis muscle and capsule of the shoulder joint and inferiorly by the teres major muscle. It is confined medially by the long head of the triceps and laterally by the surgical neck of the humerus [10]. It contains loose connective tissue, fat, veins, AXN, and the PHCA.

Typically, conservative measures such as physical therapy and physical activity modification are first recommended to patients [2]. Surgical decompression is considered when patients are unresponsive to conservative measures for at least six months [11]. The current study aims to analyze the various surgical approaches used in the decompression of neurovascular structures in QSS and presents the reflections and recommendations.

## Materials and methods

A pilot study was conducted from January to August during the 2020-2021 academic session involving first-year medical students in the Department of Anatomy at All India Institute of Medical Sciences (AIIMS), Rajkot. Ethical approval was not required for this study because this cadaveric study was conducted during the routine dissection sessions of first-year medical students in the Department of Anatomy at AIIMS, Rajkot.

**Figure 1:**
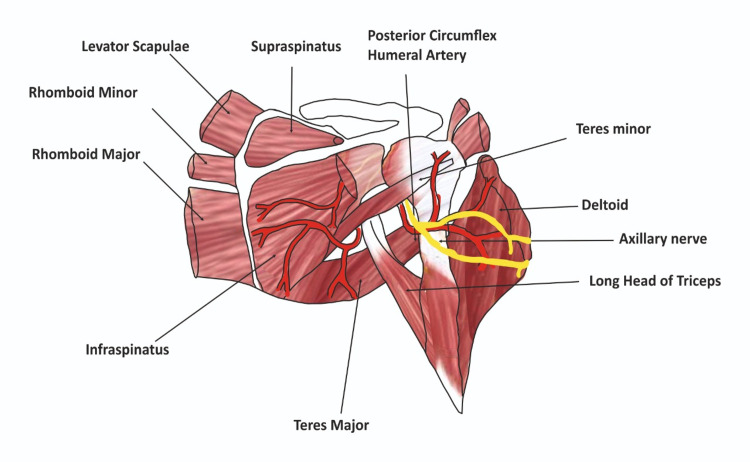
Boundaries of the quadrangular space. This figure was created by the authors.

All four boundaries of the quadrangular space are shown in Figure 1 [10].

Inclusion criteria, exclusion criteria, and sample size

The cadavers available in the Department of Anatomy, with a history of pectoral girdle pathologies or scapular region complaints, were included. Cadavers without any history of pectoral girdle pathologies or scapular region complaints were excluded. In this study, four cadavers (eight upper limbs) were used for dissection during the routine academic session.

This study will be continued over the next few academic sessions using fresh cadavers, as per the availability and feasibility, and using new dissecting personnel till a substantial amount of statistically significant data are collected to enable standardization. Therefore, this pilot study is a part of our project, and the results presented are proposed suggestions.

Parameters such as anatomical ease of identifying involved neurovascular structures, the technical expertise required to execute the procedure, probability of injury to neurovascular structures, probability of postoperative fibrosis and other complications, and the time duration required to complete the procedure were studied. The grading system was determined for every parameter studied. The anterior and posterior surgical approaches involved in the decompression procedures were confirmed and conducted on the cadavers by residents who were assisted by the faculty from the Orthopedic and Anatomy departments [12]. The observations noted by the faculty during the sessions were analyzed and documented.

Newer approach

**Figure 2:**
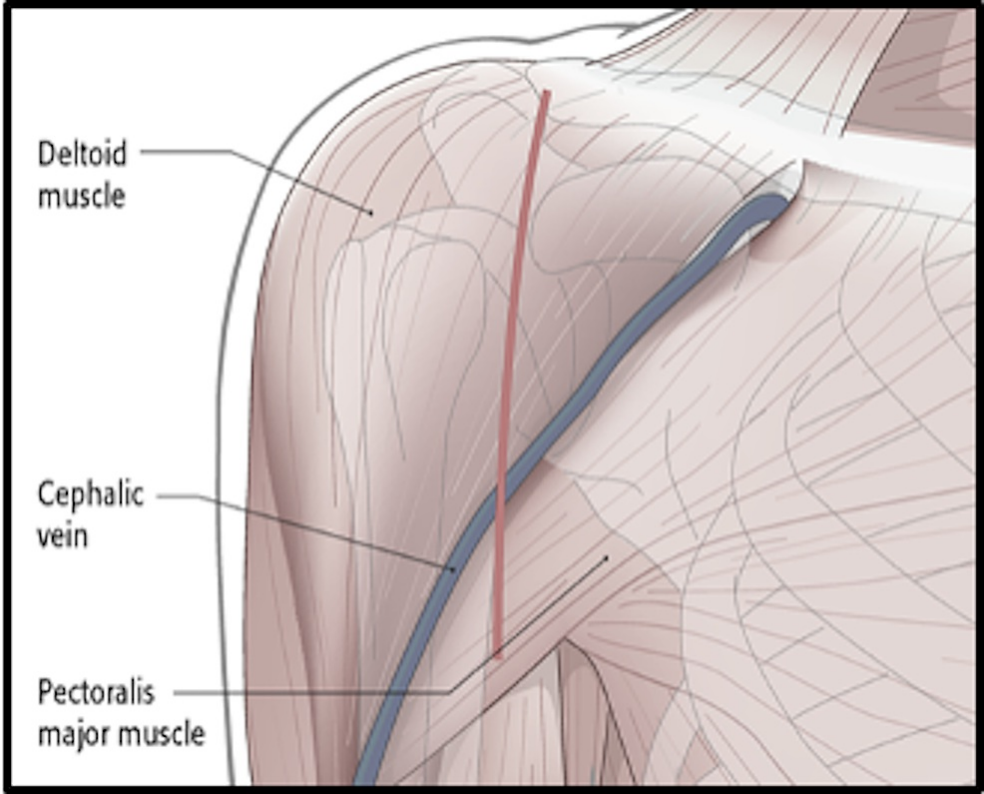
Surgical incision made in the anterior (deltopectoral) approach. Image available from https://surgeryreference.aofoundation.org/orthopedic-trauma/adult-trauma/scapula/approach/deltopectoral-approach [12].

The anterior approach, which is the preferred approach for shoulder arthroplasty, is proposed for QSS. In this approach, cadavers were placed in a supine position with the right-sided arm in a 90-degree abduction. On the right side, using the bony and surface landmarks (acromion, clavicle, coracoid process, deltoid), a 12 cm long incision was made extending from the lateral margin of the coracoid process toward the proximal humeral shaft close to the deltoid tuberosity [12]. The incision made for performing the anterior approach and the bony landmarks is shown in Figure 2.

**Figure 3:**
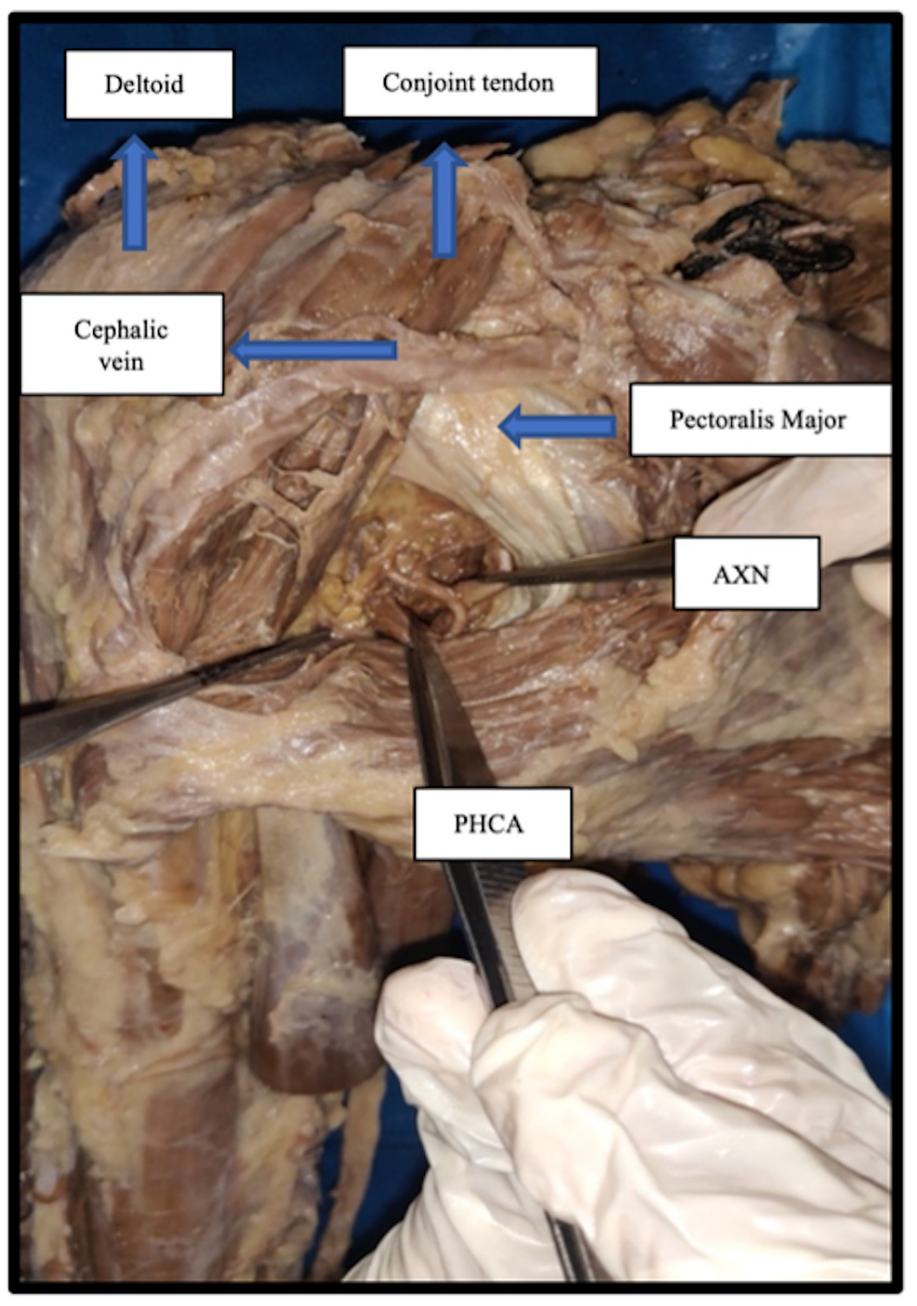
The anterior approach showing the quadrangular space between the deltopectoral groove along with the contents (AXN and PHCA) passing through it. AXN: axillary nerve; PHCA: posterior humeral circumflex artery

The residents exposed and incised the clavipectoral membrane within the deltopectoral groove. After retracting the deltoid muscle laterally and the conjoint tendon medially, the subscapularis muscle was exposed. The AXN was identified over the surface of the subscapularis muscle and was traced close to its lower border. The lower border of the subscapularis muscle is the most critical point as it is related intimately to the teres minor belly inferiorly. The skin incision was then extended below, and the lower border of the teres minor was identified. Then, using blunt dissection, the quadrangular space was traced by inserting a fingertip horizontally forward along the plane of the lower border of the teres minor. After identifying the quadrangular space, the contents of the quadrangular space can be dissected by blunt dissection. The teres major muscle need not be exposed, and by using blunt dissection, the contents of the quadrangular space (AXN and PHCA) can be identified and tracked within the quadrangular space. The quadrangular space and its contents can be cleared of any fibrous strands or adhesions (Figure 3). No muscle is cut in this approach [12]. The dissected boundaries of the quadrangular space and its contents approached through the anterior approach are shown in Figure 3.

Currently preferred approach

**Figure 4:**
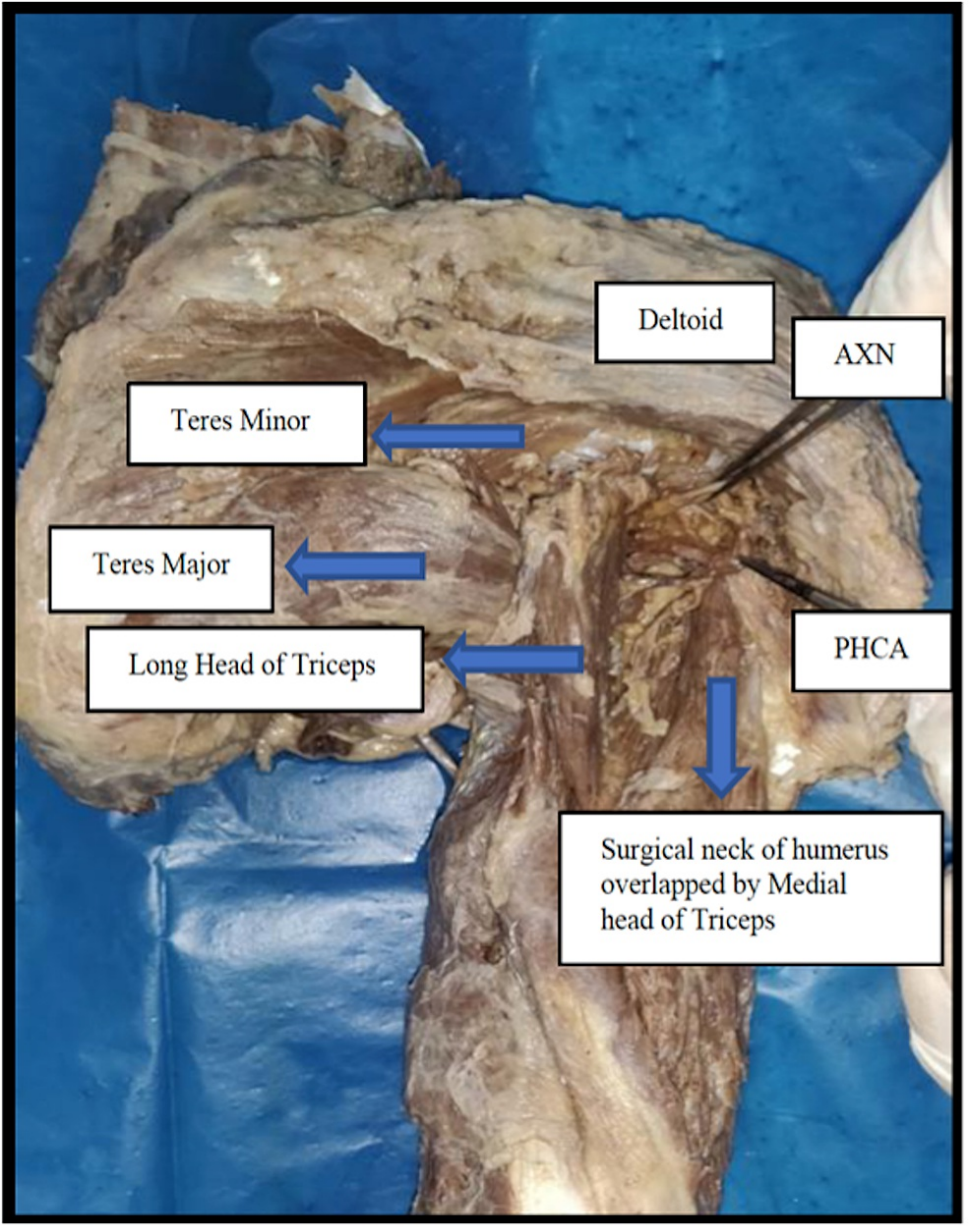
Posterior approach to the axilla showing the quadrangular space along with the contents (AXN and PHCA) passing through it. AXN: axillary nerve; PHCA: posterior humeral circumflex artery

The contralateral left-sided axillae of the cadavers were approached posteriorly, during which the cadavers were placed in a lateral decubitus position, and a longitudinal incision of approximately 4 cm was made over the posterior shoulder. The posterior border of the deltoid was secured and reflected superolaterally to reveal the underlying fat within the quadrangular space between the teres minor and the teres major. The axillary nerve and the posterior circumflex humeral vessels were then palpated as they exit the quadrangular space, following which the quadrangular space was identified and secured (Figure 4) [13]. The dissected boundaries of the quadrangular space and its contents approached through the posterior approach are shown in Figure 4.

## Results

All of the four cadavers (eight upper limbs) assigned for dissection during the routine academic session were used in the study. The anterior (deltopectoral) surgical approach was conducted on two cadavers (four upper limbs) by two residents and supervised by two faculty members. Similarly, the posterior approach was performed on the other four upper limbs by another two residents and supervised by two faculty members. A total of 25 students were divided into the two groups who observed the procedures.

Parameters such as anatomical ease of identifying involved neurovascular structures, the technical expertise required to execute the procedure, probability of injury to neurovascular structures, probability of postoperative fibrosis and other complications, and time duration required to complete the procedure were noted by the dissecting residents and faculties. These parameters were then assessed and compared between both the procedures. Table 1 enumerates all the parameters studied while performing both the approaches and presented the grading system.

**Table 1:** The grading system determined for every parameter studied.

Name of the parameter studied/observed	Grades given as a score of 10				
	Two points	Four points	Six points	Eight points	Ten points
Anatomical ease of performing the procedure	Significantly easy +	Easy ++	Average +++	Difficult ++++	Significantly difficult +++++
Requirement to incise any structure, e.g., muscle, vessels, etc.	No requirement to incise any structure at all (Nil)			Requirement to incise either one or more neurovascular structures (Yes)	
Anatomical ease in identifying involved neurovascular structures	Very easy +	Easy ++	Average +++	Difficult ++++	Very difficult +++++
Time duration required to complete the procedure	Less than 10 minutes		10–15 minutes	More than 15 minutes	
Technical expertise required to execute the procedure	Minimal +	Less ++	Average +++	High ++++	Significant +++++
Probability of injury to neurovascular structures	Minimal+	Less ++	Average +++	High ++++	Significant +++++
Probability of postoperative fibrosis and other complications	Minimal +	Less ++	Average +++	High ++++	Significant +++++

Table 2 shows the comparison of both the anterior and posterior approaches performed based on all the parameters studied while performing both the approaches and presents the grades assigned to each parameter.

**Table 2:** Assessment of parameters of both the approaches studied.

Parameters on which the approach is tested	Anterior/Deltopectoral approach (Score: 10)	Posterior approach (Score: 10)
Anatomical ease of performing the procedure	06 (+++)	08 (++++)
Requirement to incise any structure, e.g., muscle, vessels, etc.	Nil	Yes
Anatomical ease in identifying involved neurovascular structures	06 (+++)	08 (++++)
Time duration required to complete the procedure	More than 15 minutes	Less than 10 minutes
Technical expertise required to execute the procedure	high++++)	Less (++)
Probability of injury to neurovascular structures	06 (+++)	08 (++++)
Probability of postoperative fibrosis and other complications	Minimal (+)	Significant (+++++)

## Discussion

Before discussing the various surgical approaches used for decompression in QSS, and the studies conducted on it, an overview of the functional anatomy of AXN and PCHA needs to be discussed.

Functional anatomy of the axillary nerve

The AXN is found anterior to the subscapularis, posterior to the brachial artery, and lateral to the radial nerve. At the inferior aspect of the subscapularis, the AXN runs posterior, close to the joint capsule, passing through the quadrangular space with the PHCA, sandwiched between the lateral and long heads of the triceps muscle. The anterior branch of the AXN innervates the deltoid muscle, whereas its posterior branch innervates the teres minor and the deltoid. In addition, the posterior branch innervates the skin over the inferior two-thirds of the deltoid muscle at its posterior aspect [14].

Functional anatomy of the posterior humeral circumflex artery

The PHCA enters the posterior scapular region by passing through the quadrangular space. It divides into anterior and posterior branches within the quadrangular space and wraps antecedently around the surgical neck of the humerus to give blood force to the superior, inferior, and side portions of the humeral head, the glenohumeral joint, and the surrounding shoulder muscles [15,16].

In their study, Cahill and Palmer (1983) proposed a posterior approach in which a transverse incision was taken parallel and just inferior to the spine of the scapula, and curved it inferiorly over the posterior aspect of the humerus. Then, the deltoid was removed from the spine of the scapula. The teres minor was detached at its insertion into the rotator cuff and reflected medially. Decompression of the quadrangular space was done by blunt and sharp dissection. They reported satisfactory results in 16 out of 18 patients using this approach [13]. Similar observations were reported while conducting the posterior approach presented in this study.

Pitfalls of this technique

There are some pitfalls of this technique. Removal of the deltoid and teres minor can result in excess bleeding intraoperatively [13]. Division of the teres minor can weaken the rotator cuff and the lateral arm rotation [13]. The postoperative wide scar may itself compress the neurovascular bundle [13]. Furthermore, postoperative chronic pain and the formation of poor quality tissue can be observed [17,18].

In their study, Francel et al. (1991) suggested another posterior approach in which a vertical or S-shaped incision was made on the point of maximum tenderness, that is, the quadrangular space and skin flaps raised to expose the inferior border of the deltoid. The deltoid was retracted superiorly after incising the deltoid fascia and the teres muscle bellies were exposed. The fascia between the teres muscle bellies was opened, and the quadrilateral space was entered. The deltoid and teres minor muscles were not divided in this technique. The AXN and the PHCA were identified and isolated. Nerve stimulation and the motor response of the teres minor and deltoid were confirmed. Fibrous bands were divided and the space was decompressed by finger insertion [18]. Similar observations were noted while conducting this posterior approach in the present study.

Advantages of this technique

There are several advantages of this technique. The intact deltoid and teres minor can reduce bleeding, and quick postoperative shoulder movement is possible [13]. Fibrous atrophy of the deltoid can be prevented [13]. Moreover, the postoperative scar is smaller [13].

Andermahr et al. in their study mentioned the (anterior) deltopectoral approach which is usually or regularly used for almost any shoulder fracture treatment and is often the preferred approach, especially in anterior glenoid fractures [12]. A similar approach was followed by the authors in the present study, and similar observations were noted (Table 2).

Feigl et al. (2018) also used the (anterior) deltopectoral approach to visualize the AXN anteriorly. In their cadaveric study, in 91 out of 92 limbs, AXN was identified at the inferolateral border of the subscapular muscle to enter the quadrangular space. In this approach, the insertion of the subscapular muscle at the lesser tubercle defines the roof of the space [19]. We also noted similar observations in this study.

Reflections and recommendations

According to anatomists, the posterior approach was a comparatively easier and quicker method (dissection-wise) to dissect the quadrangular space, thereby identifying the AXN and PHCA. However, it is associated with postoperative complications related to the innervation and detachment of the deltoid muscle. The functional deficits associated with these problems often result in chronic pain. During the cadaveric study, anatomists observed and orthopedics agreed that the anterior (deltopectoral) approach, which is the preferred approach for shoulder arthroplasty and proximal humeral pathologies, can be effectively used for decompression in QSS. The anterior approach can easily be combined with an ultrasound-guided anesthetic block to the AXN. We suggest more cadaveric studies to facilitate anatomists and surgeons with increased opportunities to practice and revise older and newer surgical approaches.

Limitations

This study was conducted on only a few cadavers. However, this study will be followed up every academic year with more cadavers to obtain generalizable findings.

## Conclusions

According to the observations of this pilot study, the anterior approach is technically easier to perform and can be used for decompression in QSS. Moreover, this approach can easily be combined with an ultrasound-guided anesthetic block to the AXN.

## References

[1] Miller RH, Azar FM, Throckmorton TW (2013). Shoulder and elbow injuries. Campbell’s Operative Orthopaedics.

[2] van de Pol D, Kuijer PP, Langenhorst T, Maas M (2012). High prevalence of self-reported symptoms of digital ischemia in elite male volleyball players in the Netherlands: a cross-sectional national survey. Am J Sports Med.

[3] Cormier PJ, Matalon TA, Wolin PM (1988). Quadrilateral space syndrome: a rare cause of shoulder pain. Radiology.

[4] McClelland D, Hoy G (2008). A case of quadrilateral space syndrome with involvement of the long head of the triceps. Am J Sports Med.

[5] Reutter D, Hunziker R, Husmann M (2010). Computed angiogram of the upper extremities for diagnosing a rare cause of brachial arterial embolism: the 'Pitcher Syndrome'. Eur Heart J.

[6] Chautems RC, Glauser T, Waeber-Fey MC, Rostan O, Barraud GE (2000). Quadrilateral space syndrome: case report and review of the literature. Ann Vasc Surg.

[7] Brown SA, Doolittle DA, Bohanon CJ (2015). Quadrilateral space syndrome: the Mayo Clinic experience with a new classification system and case series. Mayo Clin Proc.

[8] Brown DL, Chung KC (1999). Quadrangular space syndrome associated with superficial radial sensory neuropathy. Ann Plast Surg.

[9] Jackson MR (2003). Upper extremity arterial injuries in athletes. Semin Vasc Surg.

[10] Snell RS (2011). Clinical anatomy by regions. https://www.yumpu.com/en/document/view/55653009/snells-clinical-anatomy-by-regions-9th-edition.

[11] Manske RC, Sumler A, Runge J (2009). Quadrilateral space syndrome. Hum Kinet.

[12] Jonas Andermahr, Michael McKee, Diane Nam. (2021). Deltopectoral approach to the scapula. https://surgeryreference.aofoundation.org/orthopedic-trauma/adult-trauma/scapula/approach/deltopectoral-approach#skin-incision.

[13] Cahill BR, Palmer RE (1983). Quadrilateral space syndrome. J Hand Surg Am.

[14] Rea P (2016). Essential clinically applied anatomy of the peripheral nervous system in the head and neck. https://www.elsevier.com/books/essential-clinically-applied-anatomy-of-the-peripheral-nervous-system-in-the-head-and-neck/rea/978-0-12-803633-4.

[15] Juneja P, Hubbard JB (2020). Anatomy, shoulder and upper limb, arm teres minor muscle. FL): Aug 22.

[16] Elzanie A, Varacallo M (2020). Anatomy, shoulder and upper limb, deltoid muscle. FL): Aug 22.

[17] Jon Warner JP, Iannotti JP, Flatow E (2005). Complex and revision problems in shoulder surgery. Chapter.

[18] Francel TJ, Dellon AL, Campbell JN (1991). Quadrilateral space syndrome: diagnosis and operative decompression technique. Plast Reconstr Surg.

[19] Feigl G, Aichner E, Mattersberger C, Zahn PK, Avila Gonzalez C, Litz R (2018). Ultrasound-guided anterior approach to the axillary and intercostobrachial nerves in the axillary fossa: an anatomical investigation. Br J Anaesth.

